# Mass envenomation by honey bee-speed thrills

**DOI:** 10.4103/0974-2700.70765

**Published:** 2010

**Authors:** N Balamurgan, S Senthilkumaran, P Thirumalaikolundusubramanian

**Affiliations:** Department of Emergency Medicine & Neurosciences, Sri Gokulam Hospital & Research Institute, Salem, India

Sir,

Bee stings are rare in metropolitan cities, freakish incidents were observed. Honey bees are unique among most hymenoptera, unlike other insects that sting; the honey bees leave their barbed stinger after stinging, and subsequently die after one sting because they disembowel themselves.[1] The stinger continues to inject the venom by a valve system even after the bee leaves the site of sting and not by external compression of the venom sac. Mass stinging[2] lead to life-threatening complications. The estimated lethal dose is approximately 20 stings/kg in most mammals. In this letter, a case of massive bee sting is presented for the successful revival and to stress the importance of fast removal of stinger.

A healthy 65-year-old male manual laborer, while cutting a tree in bare body, disturbed the adjoining honey-bee nest. He was immediately attacked by a large swarm of bees and sustained massive bee sting all over the body, and was brought to the emergency room within 30 min of attack. There was no history of chest pain, shortness of breath, vomiting, and giddiness. On examination, he was oriented and had several hundreds bee stingers over face, extremities, and torso with erythema, edema, and pain at the sting site. His vitals were stable. His systemic examination was unremarkable.

In the emergency room, he was supplemented with oxygen, intravenous fluids, corticosteroids, antihistamines, H2 blockers, opioid analgesics, and tetanus toxoid. The embedded bee stingers in his body were removed quickly by a team of doctors, staff nurses, and paramedics either by scraping or pinching them off. He was admitted for supportive care and observed for delayed toxicity to envenomation. He was stable for the next 48 h of admission and was discharged in good condition on the third day of hospitalization. No sequelae were observed after 1 month of follow-up.

Conventionally, practitioners were taught and counseled to scrape the stinger off rather than pluck it out with fingers or tweezers. It is believed that by pinching the stinger with forceps or fingers, remaining venom in an attached sac will be squeezed into the person who was stung, resulting in more envenomation but the morphology of the stinger does not support for this.[3]

Visscher *et al*.[4] demonstrated in their experiment that when stingers remain longer in the skin of the victim, the extent of weal size was greater. They also compared the scrape and pinch-removal methods and found that no difference in weal size. These observations stress the need for removal of stingers and not the technique. Therefore, successful management of mass bee sting depends on prompt recognition and early initiation of treatment including removal of the stinger as quickly as possible by scraping, and this has to be taught to medical and paramedics of all systems of medicine.
